# Smoking cessation aids and strategies: a population-based survey of former and current smokers in Norway

**DOI:** 10.1186/s12889-022-13032-z

**Published:** 2022-03-31

**Authors:** Marianne Lund, Ingeborg Lund

**Affiliations:** Department of Alcohol, Tobacco and Drugs, Norwegian Institute of Public Health, Oslo, Norway

**Keywords:** Tobacco, Smoking, Cessation, Quit attempt, NRT, Snus, E-cigarettes, Cessation apps

## Abstract

**Background:**

In Norway, tobacco consumption is equally divided between combustible (cigarettes) and non-combustible (snus) tobacco. In the process of quitting, people who smoke can choose between several smoking cessation aids and strategies based on what is available on the market or what are recommended as cessation aids. A quit attempt may be planned or unplanned and consist of a gradual decline in consumption or an abrupt quitting. This study explores smoking cessation aids and strategies used at the latest quit attempt among people who have ever smoked. How prevalent is the use of various cessation aids and strategies, and do they correlate with each other? Are there any differences in successful quits depending on the use of a specific cessation aid or strategy?

**Method:**

We used repeated cross-sectional representative surveys in Norway for 2017, 2018, 2019 and 2020. The analytic sample consists of people aged 20 years or older who have ever smoked daily, more precisely current daily smokers with at least one quit attempt (*n* = 476), and former daily smokers who quit in 2012 or later (*n* = 397). Participants answered questions on cessation aids and strategies used at their last quit attempt. Logistic regression analysis was used to estimate the associations between cessation aids and strategies and sociodemographic and smoking-related variables and successful quit attempts.

**Results:**

Fifty-six percent of people who ever smoked daily reported any use of cessation aids, and nicotine replacement therapy (NRT), snus and e-cigarettes were the most commonly used cessation aids. Snus and web/mobile use was associated with successful quits, while NRT was associated with unsuccessful quit attempts. When exclusive use was separated from the combined use of several aids, only snus was associated with successful quits.

**Conclusion:**

Snus use was found to be a “stand-alone” cessation aid, and only weakly associated with the use of other cessation aids. Further investigation of cessation aid preferences is needed, especially among smokers with little or no contact with health services and/or for whom traditional cessation aids have no appeal.

## Introduction

Daily smoking rates are at 8% in Norway, while occasional smoking rates are stable at 10% [1]. Smoking is most prevalent in the oldest age group, and increased cessation activity is needed to quickly reduce the health burden from smoking. Norway has a long tradition of snus use (low nitrosamine smokeless tobacco), with a steady increase in use among young males in recent decades. The tobacco sales volume is now equally divided between combustible and non-combustible tobacco, and daily use of snus has surpassed daily cigarette smoking, a unique situation internationally [2]. Snus has previously been identified as aiding people who smoke to quit [3–7].

The use of e-cigarettes is low, with 1% daily use and 2% occasional use, with users mainly being current or former smokers [2]. E-cigarette liquids with nicotine are still not legally sold in Norway, but the private import for personal use is permitted [2]. The final regulation of e-cigarettes is expected in 2022 and will coordinate with the Tobacco Products Directive (TPD) in the European Union (EU). In Europe, the use of e-cigarettes as smoking cessation aids increased while the use of pharmacotherapy declined in the period 2012–2017 [8]. In the US, e-cigarettes have surpassed pharmaceutical cessation aids among quit attempters [9]. E-cigarettes with nicotine have been shown to be effective for smoking cessation [10–17].

Even though tobacco regulation is considered strong in Norway, and the country ranks as number four on the Tobacco Control Scale, there is a potential for improvement in treatment to help smokers stop [18]. The Norwegian Directorate of Health provides information and cessation advice regarding smoking and snus to the public through a website and a mobile app, while health personnel are informed through a smoking cessation guideline and offered training programmes in smoking cessation. The mobile app offers daily motivational messages, quitting advice, facts about the health risks of tobacco and tracking of abstinence period [19]. In addition, all municipalities offer health promotion services, including tobacco cessation advice focusing on behavioural counselling and the use of pharmacotherapy [20].

In addition to available cessation aids, there are individual behavioural strategies that quit attempters may choose between. Most smoking cessation guidelines recommend an abrupt or “cold turkey” quitting strategy rather than gradual quitting, but the findings are inconclusive [21–23]. Furthermore, unplanned or spontaneous quit attempts are more successful than planned attempts in some studies [24, 25]. The benefit of an unplanned quit attempt may be attributable to the interaction with abrupt quitting [26].

Identification of smoking cessation methods used in the general population of smokers is vital for improving our understanding of the quitting process. We define smoking cessation methods as specific cessation aids and behavioural strategies. Using aids for smoking cessation is associated with an increased success rate [27], although most quit attempts are unaided [28–32]. In general, quit attempts have a high degree of failure. While the number of attempts needed before successful quitting is achieved is difficult to estimate, studies have reported an average number ranging from 6 to 30 attempts [33, 34].

More knowledge on the use of smoking cessation methods and strategies, and their potential effect on quit attempt outcomes, is needed to facilitate increased quit rates in the adult population of smokers. This study aims to explore smoking cessation aids and strategies used at the latest quit attempts among people who have ever smoked. How prevalent is the use of various cessation aids and strategies, and how do cessation methods correlate with each other? Are there any differences in successful quits depending on the use of specific cessation aids or strategies?

## Methods

### Design and sample

The data stem from the Norwegian Tobacco Surveys organised by Statistics Norway on behalf of the Norwegian Institute of Public Health (NIPH). Each year, a representative sample of 3000 persons aged 16–79 are drawn from a population database and contacted by telephone. The sample is drawn so that the percentage distribution of gender and (10-year) age groups is equal to the distribution in the population. Response rates lie around 58%. There is some underrepresentation of people with low education and in the age group 25–44 years [35]. Our analytical sample consists of people who have ever smoked who answered questions on smoking cessation aids and strategies used in their last quit attempt (*N* = 981). More precisely, our analytical sample consists of people who have formerly smoked daily and quit entirely in 2012 or later and people who currently smoke daily with at least one quit attempt. The time frame for successful quitting attempts among people who formerly smoked was set to 2012 or later to reduce potential problems with memory bias. Smoking cessation information was only available for the current year of quitting. We used a pooled data file for 2017, 2018, 2019 and 2020 and restricted the sample to persons aged 20 years or older. People below age 20 were excluded from analysis due to short time of smoking experience.

*Smoking status* was identified in three steps. Current smoking status was identified by an introductory question, with a follow-up question identifying people who smoked on a daily or occasional basis. Non-smokers and people who smoke on a non-daily basis were asked if they had ever smoked daily. People who never had smoked on a daily basis were excluded from the analysis, giving an analytic sample consisting of people who currently smoke or formerly smoked on a daily basis.

### Quit aids

People who smoke daily and people who formerly smoked daily were asked the question: “At your last quit attempt, did you use [cessation aid]?” listing six smoking cessation aids. The question about the use of NRT was asked in general, exemplified by patches and/or gum. The answer option was yes/no, and these binary variables were used in their original form in the analyses. In addition, we constructed a variable labelled “exclusive cessation aids”. This variable separated those who used no aids (0) from those who used one aid (snus (1), NRT (2) or e-cigarettes (3)), and those who used a combination of aids. This last category also included the exclusive use of the low-prevalence aids (medication, *n* = 12, web, *n* = 20 and health service *n* = 13).

### Quit strategies

Unplanned versus planned quit attempt was measured by asking the question: “Which of the following applies to your most recent quit attempt?” The answer options were “I did not plan the quit attempt, I just quit”, “I planned to quit later that day”, “I planned to quit one day earlier”, “I planned to quit several days before”, “I planned to quit several weeks before”, “I planned to quit several months before” and “None of the above”. The first answer option identified unplanned quitting, while the six following options defined planned attempts to quit [25]. The question “At your last quit attempt, did you reduce the number of cigarettes ahead of the quit attempt or did you quit without any reduction?” identified an abrupt versus a gradual quit strategy. The answer options were; “I reduced the number of cigarettes before quitting” and “I quit without any preceding reduction in cigarette consumption”.

### Sociodemographic and smoking behaviour questions

The current number of cigarettes per day (CPD) was asked of people who smoke every day, while people who formerly smoked were asked about their daily consumption the year before they quit successfully. Both CPD and age for daily smoking onset were used as a continuous variable in the analysis. Sociodemographic characteristics were gender, age (continuous), and education (0 = primary and secondary education, 1 = tertiary education (lower and higher university level)).

#### Analysis

We present descriptive statistics for socio-demographics, smoking behaviour and cessation aids and strategies for the study sample of people who ever smoke daily, and separately for people who smoke daily currently and formerly, respectively. For continuous variables, we present means and standard deviation (SD) and proportions for categorical variables. Comparisons between smoking status groups were analysed by t-test and Chi-square tests. A correlation matrix using Kendall’s tau give an overview of the association between the cessation aids and strategies.

We used smoking cessation aids and strategies as dependent variables in five separate logistic regression analyses to capture users’ characteristics (we omitted cessation aids with a low prevalence of use). The association between cessation aids and successful quits (people who smoked formerly) and unsuccessful quits (people who currently smoke) were analysed in three different logistic regression models, all using successful/unsuccessful quits as the dependent variable. The first model uses the binary cessation aids as independent variables, the second model the constructed “cessation aids” identifying exclusive use as independent variables, and model 3 uses cessation strategies as independent variables. The logistic regression models present crude odds ratio (OR) and adjusted odds ratios (AOR) with corresponding 95% confidence intervals (CI). All analyses were done in Stata version 16.

## Results

### Descriptive statistics of quit attempters

As shown in Table 1, 53% of the study sample were male, the mean age was 50 years, and 79% had primary or secondary educational attainment. The mean age for smoking onset was 18 years, and the average CPD was 13. Overall, 44% reported no use of any of the listed smoking cessation aids, with a higher proportion among unsuccessful quitters (47%) than successful quitters (40.5%). The most common cessation aids were NRT, snus and e-cigarettes. A planned quit attempt was more common than an unplanned quit attempt, and abrupt quitting was more common than a gradual reduction in consumption. No gender difference was observed in unsuccessful versus successful quits. However, unsuccessful quit attempters were more often older people with low educational attainment and fewer cigarettes per day than successful quit attempters.

**Table 1:** Descriptive statistics. People who ever smoke daily, currently smoke daily, and formerly smoked daily, 20 years or older. Pooled data file 2017–2020. *n* = 874

	People who ever smoke daily (A + B) % (n)	People who currently smoke daily (unsuccessful quitters, A)^a^ % (n)	People who formerly smoked daily (successful quitters, B)^b^ % (n)	Pearson’s Chi-square/t-test
Total n	874	476	398	
Gender				
Male	53.0 (463)	51.9 (247)	54.3 (216)	
Female	47.0 (411)	48.1 (229)	45.7 (182)	0.483
Age, years, mean (SD)	50.3 (14.2)	51.7 (13.4)	48.7 (15.0)	0.002
Education				
Primary/Secondary	78.6 (667)	81.8 (377)	74.7 (290)	
Tertiary	21.4 (182)	18.2 (84)	25.3 (98)	0.013
Age at onset of smoking, mean (SD)	17.9 (5.4)	17.8 (5.5)	18.0 (5.3)	0.695
Cigarettes per day, mean (SD)	13.1 (7.4)	11.7 (6.3)	14.8 (8.2)	0.000
No method used	44.1 (385)	47.2 (224)	40.5 (161)	0.098
Used snus	18.6 (162)	12.2 (58)	26.1 (104)	0.000
Used NRT	18.7 (163)	21.7 (103)	15.1 (60)	0.013
Used prescription medication	9.2 (80)	10.7 (51)	7.3 (29)	0.078
Used e-cigarettes	17.2 (150)	17.7 (84)	16.6 (66)	0.667
Used health service	9.6 (84)	11.0 (52)	8.0 (32)	0.147
Used web/mobile	9.2 (80)	6.7 (32)	12.1 (48)	0.007
Unplanned (vs planned)	35.5 (309)	32.3 (153)	39.3 (156)	0.031
Abrupt (vs gradual)	71.7 (626)	68.2 (324)	75.9 (302)	0.012

^a^Current daily smokers with at least one quit attempt who answered questions on the use of cessation aids at their latest quit attempt

^b^Former daily smokers who quit in 2012 or later, and who answered questions on the use of smoking cessation aids at their latest (and successful) quit attempts

### Characteristics of users of cessation aids and strategies

Those who reported using snus as a smoking cessation aid were mainly young males. In contrast, NRT was used by older females and with high CPD (Table 2). The use of e-cigarettes as a cessation aid was also more common among women with low educational attainment and a high CPD. Unplanned and abrupt quit strategies were more likely among males than among females.

**Table 2:** Logistic regression analysis of the most common cessation aids and strategies as dependent variables (five separate models) and sociodemographic and smoking behaviour as independent variables. Adjusted odds ratio (AOR) and 95% confidence intervals (CI). *n* = 864

	Snus AOR	NRT AOR	E-cigarettes AOR	Unplanned AOR	Abrupt AOR
Total n	864	864	864	862	864
Gender					
Male	Ref.	Ref.	Ref.	Ref.	Ref.
Female	0.31 (0.20–0.47)	1.96 (1.36–2.82)	1.94 (1.33–2.82)	0.68 (0.51–0.91)	0.61 (0.45–0.83)
Age (continuous)	0.94 (0.93–0.96)	1.02 (1.01–1.04)	0.99 (0.98–1.00)	0.99 (0.98–1.00)	1.00 (0.99–1.01)
Education					
Primary/Second.	Ref.	Ref.	Ref.	Ref.	Ref.
Tertiary	1.24 (0.77–1.99)	1.16 (0.76–1.76)	0.51 (0.31–0.86)	0.84 (0.58–1.20)	1.24 (0.85–1.82)
Age at onset of smoking (continuous)	0.97 (0.92–1.02)	0.98 (0.95–1.02)	0.98 (0.94–1.02)	1.01 (0.99–1.04)	0.99 (0.97–1.02)
Cigarettes per day (continuous)	1.00 (0.98–1.03)	1.02 (1.00–1.04)	1.03 (1.01–1.06)	0.99 (0.97–1.01)	1.01 (0.99–1.04)

The correlation matrix (Table 3) shows that the use of snus as a cessation aid was negatively and significantly correlated to NRT, cessation medication and using health services, and not correlated to any other cessation aids. NRT was positively correlated to web/mobile but negatively correlated to spontaneous and abrupt quitting strategies. Spontaneous and abrupt quitting were negatively and significantly correlated to all cessation methods except snus.

**Table 3:** Nonparametric bivariate correlation (Kendall’s tau). Ever daily smokers aged 20 years or older, pooled data file 2017–2020, *n* = 870

	Snus	NRT	Medication	E-cigarettes	Health service	Web/mobile	Unplanned	Abrupt
Snus	1							
NRT	−0.106*	1						
Medications	−0.090*	0.042	1					
E-cigarettes	− 0.052	0.041	0.003	1				
Health service	−0.076*	0.064	0.556*	−0.025	1			
Web/mobile	0.033	0.154*	0.050	−0.007	0.125*	1		
Unplanned	0.037	−0.120*	−0.127*	− 0.081*	−0.128*	− 0.069*	1	
Abrupt	0.003	−0.152*	−0.154*	− 0.135*	−0.175*	− 0.074*	0.246*	1

^*^p < 0.05

### Associations between cessation methods and successful quits

Table 4 presents the bivariate and adjusted associations of each smoking cessation aid with the outcome variable successful vs unsuccessful quits. Snus and web/mobile use were the only cessation aids that were positively and significantly associated with successful quits in crude and adjusted models (model 1). The use of NRT was negatively and significantly related to successful quits, and there was no significant relationship between the other cessation aids and the outcome of successful quits. When contrasting exclusive use of cessation aids with unassisted quitting, only snus use was positively and significantly related to successful quits (Table 4, model 2). High educational attainment and a high level of CPD were significantly associated with successful quits (results not shown).

**Table 4:** Logistic regression models. Crude and adjusted odds ratio for successful versus unsuccessful quits by use of cessation methods and strategies

	Crude OR (95% CI) *n* = 871–873^1^	Adjusted model^2^ AOR (95% CI) *n* = 864
Model 1: Smoking cessation aids		
Snus	2.54 (1.78–3.62) ***	2.39(1.60–3.55) ***
NRT	0.64 (0.45–0.91) *	0.57 (0.39–0.85) **
Medication	0.65 (0.41–1.05)	0.72 (0.39–1.33)
E-cigarettes	0.93 (0.65–1.32)	0.86 (0. 58–1.26)
Health service	0.71 (0.45–1.13)	0.81 (0.44–1.50)
Web/mobile	1.90 (1.19–3.03) **	2. 17 (1. 29–3.66) **
Model 2: Exclusive smoking cessation aids		
No aids used (*n* = 385)	Ref.	Ref.
Snus only (*n* = 110)	2.34 (1.51–3.62) ***	2.23 (1.39–3.58) **
NRT only (*n* = 73)	0.72 (0.43–1.22)	0.66 (0.38–1.15)
E-cigarettes only (*n* = 82)	1.46 (0.91–2.36)	1.22 (0.74–2.03)
Combination use and other^3^ (*n* = 223)	1.15 (0.83–1.61)	1.05 (0.74–1.49)
Model 3: Smoking cessation strategies		
Unplanned (vs planned)	1.36 (1.03–1.79) *	1.30 (0.96–1.76)
Abrupt (vs gradual decline)	1.47 (1.09–1.98) *	1.34 (0. 97–1.85)

^1^Number varies due to item non-response

^2^Adjusted for all variables in the model, including gender, age, education, debut age for smoking and CPD

^3^The number of smokers who used medication, health service and web/mobile exclusively were low and included in the group of combined use. 2-tailed *p* value, where *** = *p* values< 0.001, ** *p* values< 0.01, *p values< 0.05

The association between quitting strategies and successful quitting was significant in the bivariate analysis but not in the adjusted model (Table 4, model 3). Unplanned gave a higher odds ratio for successful quits than planned attempts. Abrupt quit attempts gave a higher odds ratio for successful quits than a gradual decline in cigarette consumption.

## Discussion

More than half of ever-smokers with a quit attempt reported use of any cessation aid. Snus, NRT and e-cigarettes were the most prevalent cessation aids in the last quit attempt. Planned and abrupt quitting was preferred, and snus and web/mobile were the cessation aids related to successful quits. Snus stood out by its user profile and was the aid least often combined with other cessation methods.

Our findings that successful quits were strongly related to the use of snus are likely related to the fact that Norway has a long tradition of snus use. Snus has now replaced cigarette smoking as the most prevalent form of daily tobacco use in the population, particularly among young males [2, 36]. The association between using snus as a cessation aid and successful quits adds to previous findings from Norway [3, 37–39], and supports the notion that availability of snus might have facilitated a population-level decline in smoking [3, 6]. The impact of a specific cessation aid is contingent on its efficacy and how extensively it is used in the population [40].

The gender difference in snus use is not well understood, but the snus market was historically geared towards males. This changed after 2004, with general product innovation, including products aimed at younger women. The mechanisms behind the strong association between snus and successful quits are not well understood. The nicotine content and duration of use of snus may provide nicotine in an amount that is similar to cigarette smoking, avoiding or reducing nicotine withdrawal symptoms when quitting cigarettes [41]. There may also be social and cultural expectations towards the use of snus that help fill the gap from the habit of smoking. More research into outcome expectancy using snus and e-cigarettes as cessation aids among adults is needed, focusing on social as well as biological expectancies.

Based on our finding that quit attempters using snus are mainly younger males and that the use of snus was negatively correlated to the use of health services, we can assume that this group of smokers are non-treatment-seeking people who make their quit attempts outside the preventive health service. This assumption is supported by the fact that snus is not a recommended cessation aid in cessation guidelines. The experiential knowledge of people who used to smoke but switched to snus may spread in social networks, and thus snus might have become a cessation product that operates on the sidelines of official tobacco control strategies.

We found no association between e-cigarettes at the last quit attempt and successful quits, as measured in model 1. However, exclusive use of e-cigarettes indicated a positive but non-significant relationship. E-cigarettes have a short history among Norwegian users, and the nicotine liquid ban and reduced availability might mean that it is too early to draw conclusions about their role in smoking cessation. As for snus, e-cigarettes are not defined as a smoking cessation aid in Norway. The reasons for the higher probability of females using e-cigarettes in their last quit attempt compared to males are unknown. In contrast, in previous findings, males were more likely to report use of e-cigarettes as a cessation aid [42]. One mechanism could be that male quit attempters have found their preferred alternative cessation aid in snus and is less interested in e-cigarettes as cessation aids. Another potential explanation is that e-cigarettes have higher appeal and likeability among female quit attempters. Their habitual and sensory-like similarity to regular smoking and the possibility for choosing between different flavours are mechanisms that likely increased their attractiveness [43]. Findings on gender differences in e-cigarettes preferences, including flavour, is however mixed and further investigation into gender preferences for using snus and e-cigarettes is needed [44].

The lack of association between the use of NRTs and successful quits may be due to the higher average age and higher CPD levels among NRT users. Females were more likely to use NRT at the last quit attempt. Again, this may be due to the male dominance in using snus and lack of alternative nicotine options. Further research should investigate gender differences, and whether new nicotine products like tobacco-free nicotine pouches may have a stronger appeal to women in their quitting attempts.

We found an association between the use of web/mobile and successful quits, but we were not able to analyse the association between exclusive use of web/mobile and the outcome due to low numbers. The web/mobile alternative was mainly used in combination with other cessation aids like NRTs or health services. Web/app solutions for smoking cessation have a great potential for reaching out to many people who smoke, including people who are not currently in a quitting process.

We observed a tendency that both unplanned and abrupt (cold turkey) cessation strategies were associated with successful quits, although the associations were not significant in adjusted models. Previous findings indicate that unplanned and abrupt strategies are beneficial for successful quits, but the findings are not consistent. All cessation aids except snus were associated with planned and gradual decline in cigarette consumption. Whether this indicates that people who report using snus at their last quit attempt may be defined as “accidental” quitters, as suggested for those who use e-cigarettes, is not known [45]. The role of specific cessation strategies for successful quits needs to be explored further, including any potential interactions with the use of specific smoking cessation aids.

There are substantial educational differences in smoking behaviour in Norway, but the same gradient is not observed for snus use [2]. Education is a strong predictor of smoking behaviour in Norway, as it is in the European countries at large [46]. The higher share of people with low education among unsuccessful quitters in this study indicates a need for increased attention to the use of cessation aids, including new nicotine and low-risk tobacco products, to combat this difference. We found educational differences in the use of e-cigarettes at last quit attempt, where people with primary/secondary educational level were more likely to have used e-cigarettes. Reducing social inequality in smoking behaviour is a high priority, and the contribution of e-cigarettes in reducing inequality needs further attention. Our finding is in line with results from England, where use of e-cigarettes was more common among those with lower socioeconomic status than those with higher socioeconomic status among last year quitters [47].

To some degree, our results demonstrate a discrepancy between the recommended cessation aids and the aids people who try to quit smoking use. It seems like younger males have found their way out of smoking through the route of snus. It is less clear for older smokers and females whether NRT or e-cigarettes play the same role. In our study, unsuccessful quit attempters were more often older people with primary or secondary school as their highest educational level. This group of smokers may need unambiguous advice from authoritative health professionals and smoking cessation guidelines to combat smoking using a wide range of cessation aids, including alternative nicotine products. The use of health services for smoking cessation was low, and the reason for this is not known.

There are some limitations in our study. First, it presents associations between the use of smoking cessation aids and successful quits and cannot reveal any causal relationship due to our cross-sectional design. Second, the questions are retrospective, and there is always a risk of recall bias. Third, due to a lack of information on the time frame for unsuccessful quit attempts, we cannot guarantee that all attempters would have had access to the same number of cessation aids. Fourth, the number of unassisted quit attempts was based on those who did not use any of the listed cessation aids, and not based on an explicit question of no use. The list of cessation aids could also be incomplete, as self-help materials were not listed. And last, we did not have information on the type of NRT used, whether it was gum, patches, inhalator, or lozenge. This could have affected our results. In addition, we did not have information on whether e-cigarettes used at last quit attempt contained nicotine or not. However, information from population surveys reports low use of non-nicotine e-cigarettes.

## Conclusion

Snus use was found to be a “stand-alone” cessation aid, and only weakly associated with the use of other cessation aids. Further investigation of cessation aid preferences is needed, especially among smokers with little or no contact with health services and/or for whom traditional cessation aids have no appeal.

## Data Availability

The data that support the findings of this study are available from the Norwegian Centre for Research Data (NSD) but restrictions apply to the availability of these data, which were used under licence for the current study, and are not publicly available. Data are however available from the authors upon reasonable request and with permission of the Norwegian Centre for Research Data (NSD).

## Abbreviations

NRT: Nicotine replacement therapy
CPD: Cigarettes per day
TPD: Tobacco Products Directive
OR: Odds ratio
AOR: Adjusted odds ratio
CI: Confidence Intervals
SD: Standard Deviation
